# A novel gaze-controlled flexible robotized endoscope; preliminary trial and report

**DOI:** 10.1007/s00464-021-08556-1

**Published:** 2021-05-24

**Authors:** Arun Sivananthan, Alexandros Kogkas, Ben Glover, Ara Darzi, George Mylonas, Nisha Patel

**Affiliations:** 1 Imperial College Healthcare NHS Trust, London, W2 1NY UK; 2 The Hamlyn Centre for Robotic Surgery, Imperial College London, London, UK

**Keywords:** Robotic endoscopy, Eye tracking, Touchless interactions

## Abstract

**Background:**

Interventional endoluminal therapy is rapidly advancing as a minimally invasive surgical technique. The expanding remit of endoscopic therapy necessitates precision control. Eye tracking is an emerging technology which allows intuitive control of devices. This was a feasibility study to establish if a novel eye gaze-controlled endoscopic system could be used to intuitively control an endoscope.

**Methods:**

An eye gaze-control system consisting of eye tracking glasses, specialist cameras and a joystick was used to control a robotically driven endoscope allowing steering, advancement, withdrawal and retroflexion. Eight experienced and eight non-endoscopists used both the eye gaze system and a conventional endoscope to identify ten targets in two simulated environments: a sphere and an upper gastrointestinal (UGI) model.

Completion of tasks was timed. Subjective feedback was collected from each participant on task load (NASA Task Load Index) and acceptance of technology (Van der Laan scale).

**Results:**

When using gaze-control endoscopy, non-endoscopists were significantly quicker when using gaze-control rather than conventional endoscopy (sphere task 3:54 ± 1:17 vs. 9:05 ± 5:40 min, *p* = 0.012, and UGI model task 1:59 ± 0:24 vs 3:45 ± 0:53 min, *p* < .001).

Non-endoscopists reported significantly higher NASA-TLX workload total scores using conventional endoscopy versus gaze-control (80.6 ± 11.3 vs 22.5 ± 13.8, *p* < .001). Endoscopists reported significantly higher total NASA-TLX workload scores using gaze control versus conventional endoscopy (54.2 ± 16 vs 26.9 ± 15.3, *p* = 0.012). All subjects reported that the gaze-control had positive ‘usefulness’ and ‘satisfaction’ score of 0.56 ± 0.83 and 1.43 ± 0.51 respectively.

**Conclusions:**

The novel eye gaze-control system was significantly quicker to use and subjectively lower in workload when used by non-endoscopists. Further work is needed to see if this would translate into a shallower learning curve to proficiency versus conventional endoscopy. The eye gaze-control system appears feasible as an intuitive endoscope control system. Hybrid gaze and hand control may prove a beneficial technology to evolving endoscopic platforms.

**Supplementary Information:**

The online version contains supplementary material available at 10.1007/s00464-021-08556-1.

Eye gaze tracking is a technology showing great possibilities for integration into surgical and endoscopic practice. Modern eye- and head-tracking sensors may be harnessed to control robotic platforms, allowing intuitive control of complex devices; this technology may improve control during complex procedures, and enhance the learning process as well as reducing cognitive demand.

Within surgical specialties, visual gaze pattern analysis has been informative for assessing the cognitive burden associated with complex procedures [1]. Novel eye gaze-control systems are in development to control the movement of laparoscopic cameras and reduce delays or error, particularly from inexperienced operators or a human assistant [2]. Eye gaze tracking has been studied for colonoscopy and virtual colonography to determine visual gaze patterns during examination, and the resultant effects on polyp detection [3, 4].

Advanced interventional endoscopy is rapidly developing to become a subspecialty in its own right. There is a myriad of potential in advance interventional endoscopy with rates and complexity of endoluminal techniques, such as endoscopic submucosal dissection (ESD), increasing [5, 6]. Despite the growing demand for these procedures, the basic control system of the flexible endoscope has not changed beyond the standard control wheels and torque steering, despite their well-documented poor ergonomics [7]. Several emerging endoscopic approaches have been proposed to facilitate advanced therapeutic procedures, including flexible insertable instruments, endoscopically deployed operating platforms, and robotically controlled dissection instruments [8–10]. Many of these platforms require several operators, including an endoscopist to control the endoscope, apply torque and stabilise it at the site of resection. Furthermore, assistants are usually required to access and operate endoscopic tools such as resection knives, clips, haemostatic devices and to manage injection of fluid into the submucosal space. All of these platforms require hand control of either the endoscope movement, end effectors or both. This requires several people within the theatre or endoscopy room which may further increase cognitive load and distraction for the operator while performing a highly skilled and challenging technique. It also requires co-ordinated and clear instructions and communication between several people which can be challenging.

We present a novel approach in which the movement of a robotic flexible gastroscope is controlled by eye gaze tracking, without the need for the operator to touch the endoscope. The full range of manual endoscopic control is reproduced by the robotic platform, including insertion and withdrawal, rotation, tip angulation, and retroflexion. We discuss the results of the initial prototype testing in anatomical models of the upper GI tract, and the associated differences in performance, cognitive load, physical demand and ergonomic stress between a conventional endoscope and the robotic gaze-controlled endoscope.

## Materials and methods

### Robotic platform

The robotic control module for control of the endoscope is described fully elsewhere [11]. Briefly, this consists of two custom 3D-printed gears placed over the endoscopy control wheels, driven by motors (Dynamixel RX-24F, Robotis, Korea), and controlled by a separate workstation. This system was mounted to a Karl Storz 13,801 PKS gastroscope. This assembly was limited to 1.0*N*m torque at the tip and an angular velocity of 50 rpm, ensuring margins of power and speed were well within those required for safe and effective endoscope control [12].

**Fig. 1:**
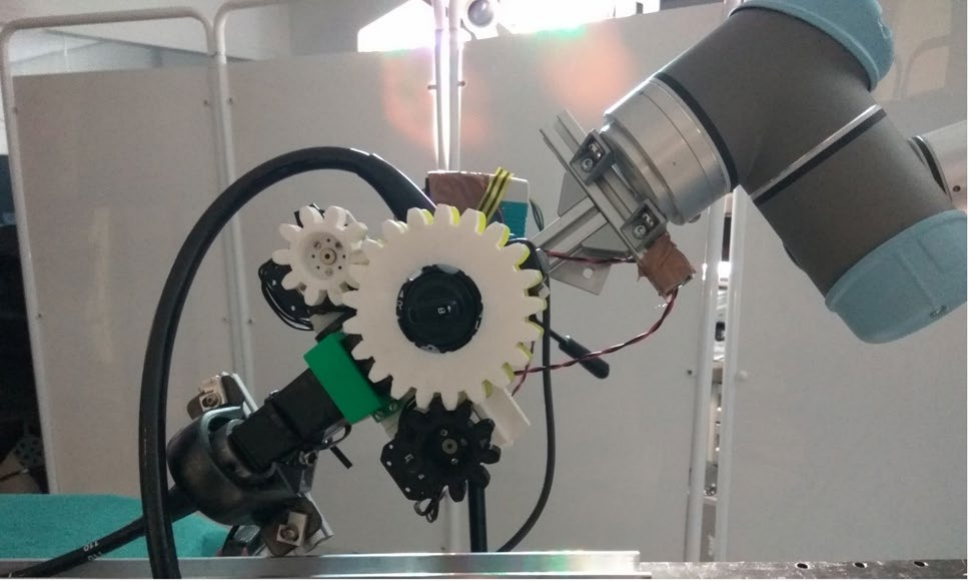
The fully robotised system

The control module and endoscope were then mounted to a robotic arm (Universal Robotics UR5) able to move in six directions and the shaft of the endoscope aligned to a guiderail to support the flexible portion of the endoscope while outside the training model (Fig. 1).

### Gaze control

**Fig. 2:**
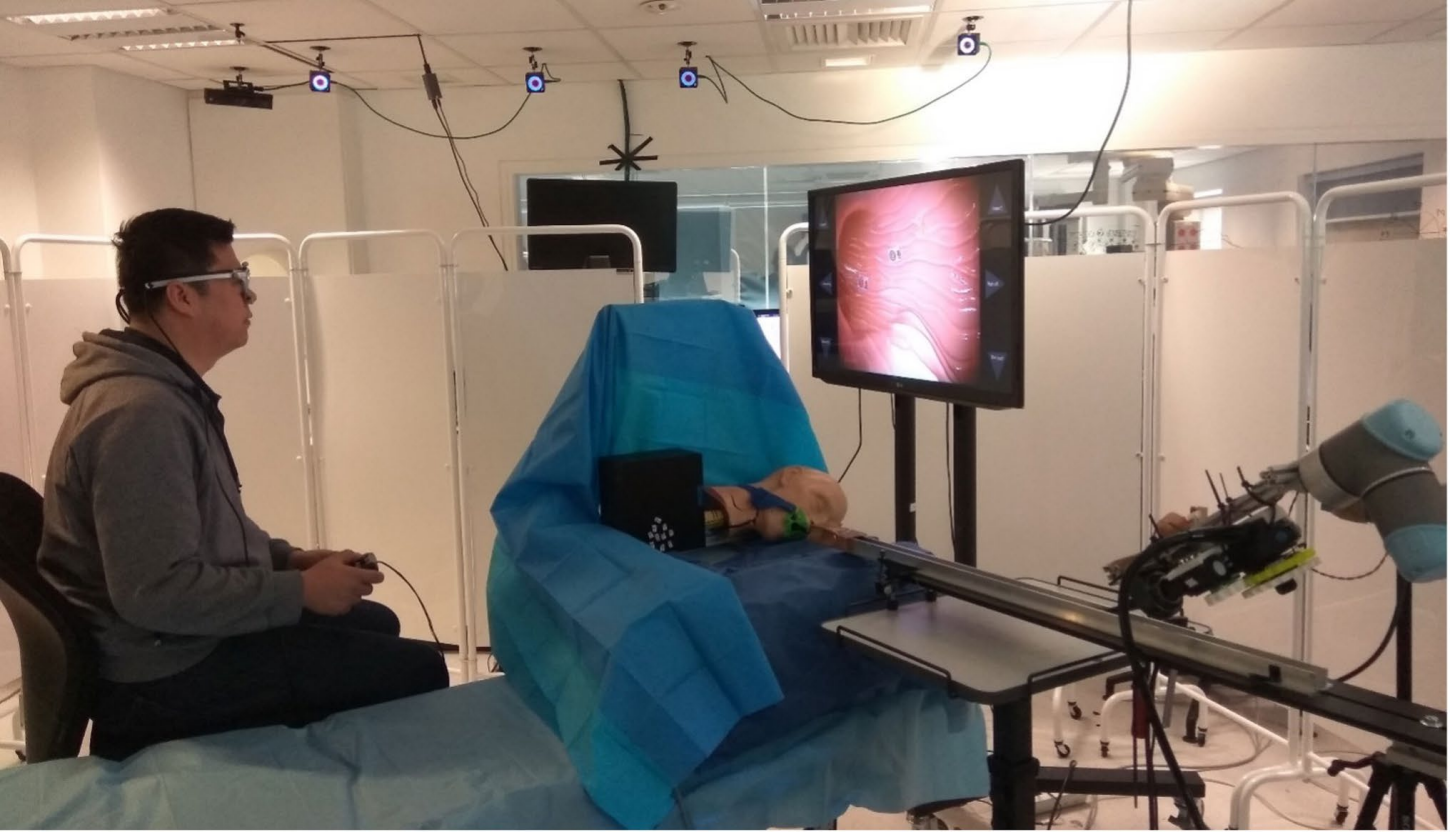
The eye gaze platform in use [20]

The primary controls were the operator’s head position and direction of gaze on a screen; these were estimated based on a free view 3D gaze-contingent framework developed by Kogkas et al. [13, 14]. This system incorporates the use of eye-tracking glasses (SensoMotoric Instruments GmbH) with an RGB-D camera (Microsoft Kinect v2) for 3D reconstruction of the environment and six reflective spheres mounted on the glasses with a four-camera motion capture system (OptiTrack^™^ Prime 13 Cameras, NaturalPoint, Inc.) for head tracking. The use of this system is shown in Fig. 2.

Further optional controls were provided by a small handheld two axis joystick connected via an Arduino Uno microcontroller. The view from the endoscope was displayed on a 42-inch LG monitor at 1920 × 1080 resolution. A Windows 10 PC was used for eye gaze and motion capture data acquisition; Ubuntu 14.04 was used for all other modules.

### User interface

Each operator sat approximately two metres from the screen, in an ergonomically neutral position wearing the eye gaze tracking glasses. The joystick was held in one or both hands according to operator preference.

Endoscope tip angulation was controlled by the eye gaze point of regard in relation to the centre of the display through a gaze-contingent closed loop velocity controller. This resulted in movement of the tip of the endoscope, and associated field of view, in the direction of the operator’s gaze.

Endoscope rotation was controlled by tilting of the head (exceeding ± 20 degrees). This was chosen to minimise limb and finger movement and to reflect the natural urge of head rotation of the endoscopist when reorienting an endoscope. When initiating rotation, the system produced an audible alert to the operator of “left” or “right”. When head orientation returns to the allowable range (± 20 degrees), the endoscope rotation stopped, and an audible alert “straight” is produced.

**Fig. 3:**
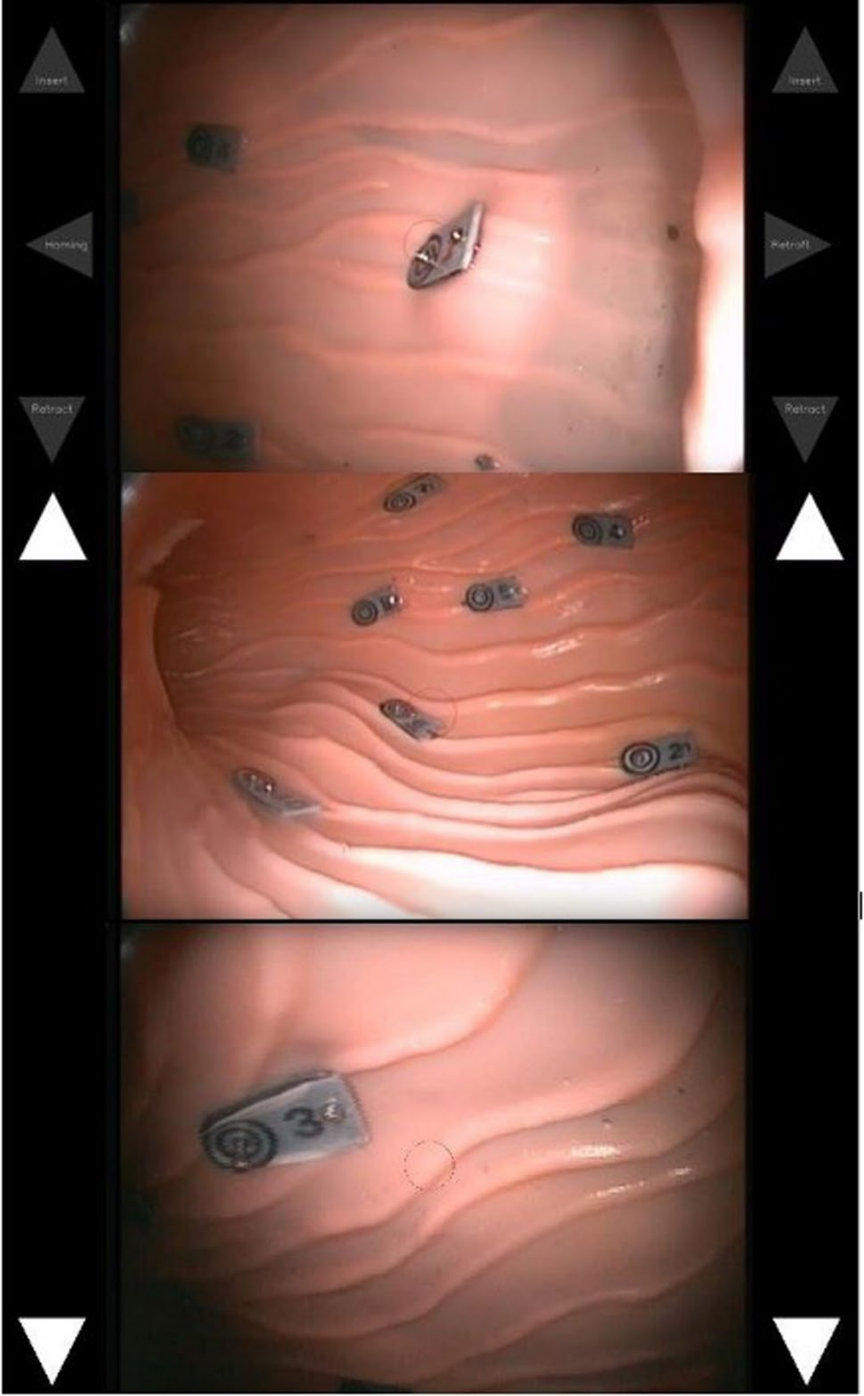
The graphical user interface of the system. Top: the view while the system is paused. Joystick control options can be seen beside the endoscopic view. Middle: The view during insertion, including graphical indicator, and Bottom: the view during withdrawal

Insertion and withdrawal were controlled by pushing up or down on the joystick controller. Corresponding up or down arrows are shown on the screen with audible “in” and “out” alerts. Retroflexion was initiated by holding the joystick controller to the right for one second or longer (audible alert: “retroflexion”). A press of a button on the joystick paused the movement of the endoscope (audible alert: “pause”), holding it in position until a further press (audible alert: “un pause”). The graphical user interface is shown in Fig. 3.

A nine-point calibration process was completed by each operator prior to using the gaze-control system. This is performed with the head in a neutral position by directing gaze at a set of fixed points on the screen in sequence. Micro-saccadic eye movements are filtered out by distinguishing high velocity eye movements (greater than 36 degrees of visual angle per second) and ‘dwell’ times of less than 0.2 s.

As an additional safety measure, the movement of the robotic system was automatically paused if the operators gaze was directed outside the field of the display and when gaze tracking was lost. In these way only purposeful eye movements control steering.

### Study design

This was a feasibility study to establish the capabilities of a novel gaze-controlled robotic flexible endoscope in a simulated environment. As a comparator, a conventionally- controlled endoscope was used to complete the same tasks. Eight endoscopists and eight non-endoscopists (no previous gastrointestinal endoscopy experience) participated providing written consent for the data to be utilised.

Endoscopists were defined as gastroenterologists who had achieved certification to perform endoscopy independently by the UK Joint Advisory Group Endoscopy Training System. This requires attendance at a course, a minimum of 300 observed procedures, and 20 examined procedures. The age range of the endoscopist group was between 32 and 51 years. Non-endoscopists were defined as those who had never performed an endoscopy before consisting of seven non-clinical scientists and one ENT surgeon. The age range of the non-endoscopist group was between 24 and 42 years.

No participants had prior experience of the robotic system. All participants underwent a standardised familiarisation process and then used both the eye gaze system and a conventional endoscope to identify ten targets in two simulated environments: a sphere and an upper gastrointestinal (UGI) model.

The two benchtop tasks to assess the efficiency, ergonomics and stability of the device were as follows:

- Task 1 was performed using a plastic spherical cavity of diameter 20 cm and with a 11 mm circular port. The starting position of the endoscope tip was 1 cm inside the port. Ten numbered paper targets were distributed on the internal wall, in differing positions and orientations. Each participant attempted to find the targets in sequence, orient the endoscopic field of view such that the target was ‘upright’, and obtain a central close view in this orientation. A circle with 400-pixel diameter was drawn on the centre of the display to define the central view. The target was successfully identified once it was placed within this circle. The tenth target required retroflexion of the endoscope tip to be adequately visualised.
- Task 2 was performed in an anatomical model of the upper GI tract and head (The Chamberlain Group, MA, USA). The inside of the stomach contained ten numbered plastic targets. The starting position of the endoscope tip was in line with the incisors. Each participant attempted to intubate the oesophagus, insert the endoscope to the stomach, and identify each of the targets in sequence, visualising them with a central, close view (within the 400-pixel diameter circle).

**Fig. 4:**
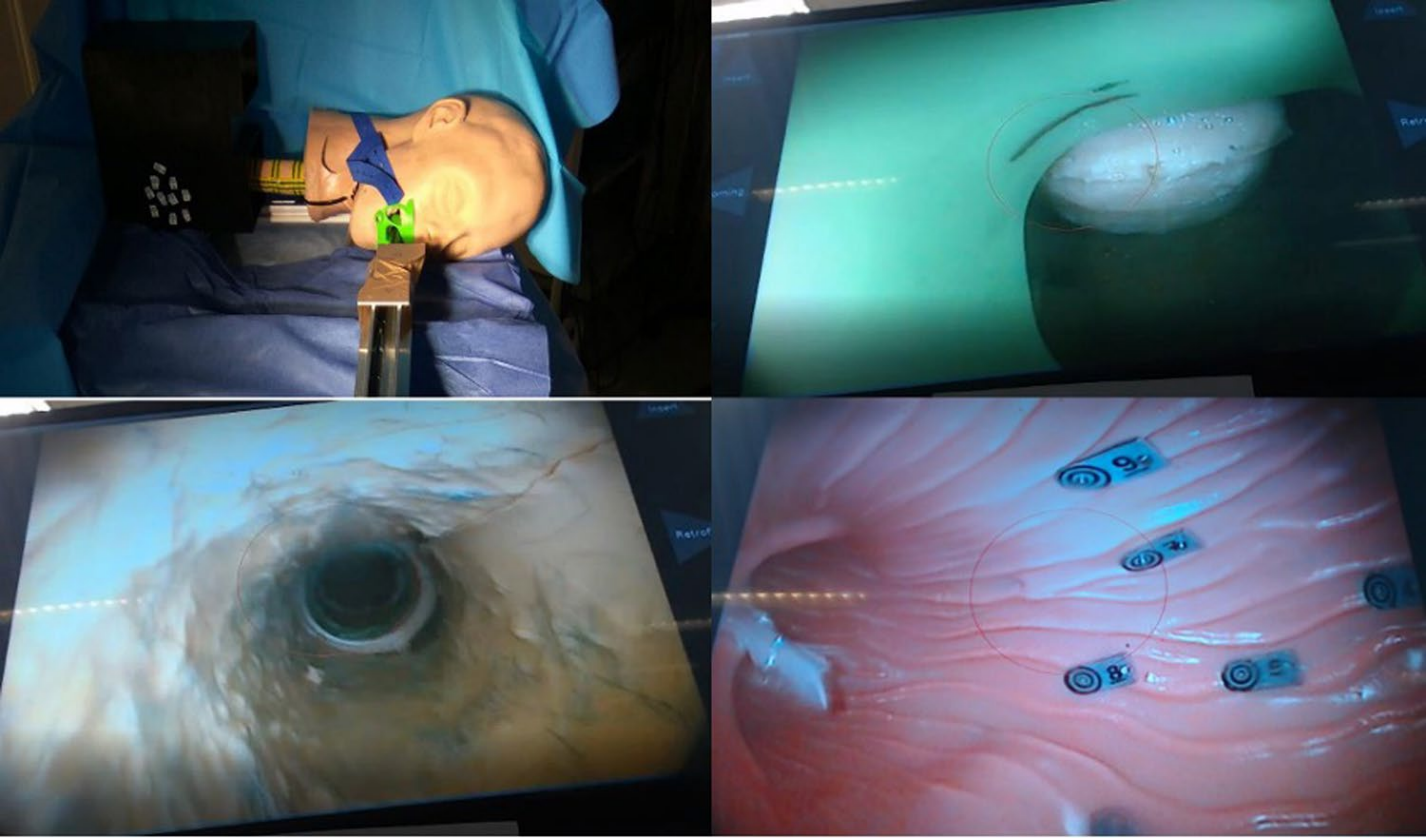
The benchtop experimental environment [20]

The benchtop experiments are displayed in Fig. 4.

Participants were randomised to initial control method used. Familiarisation for each control method took place before Task 1 of each method. This was standardised with a written set of instructions read by participants for each device and a demonstration by the instructor (NP and AK). This was followed by 10 min of use of the device on a silicon oesophageal model with practice targets on a flat surface. This familiarisation task was separate to Task 1 and 2.

Each participant completed both tasks using one control method, before changing to use the other method and repeating the tasks. Each task was completed once with each method. Task 2 always followed Task 1. Feedback was collected after the use of each method to avoid recall bias.

### Outcomes

#### Objective outcome measures

The time taken to find each target and the overall task completion times were recorded to the nearest second.

#### Subjective feedback

Subjective feedback was collected from each participant on task load, acceptance of technology and ergonomic design, directly after the tasks were completed. Task load was assessed using the multidimensional rating scale NASA-TLX (Task Load Index) [15], a ten-point visual analogue scale to assess cognitive workload (Appendix Fig. 10) which has been validated in both diagnostic and therapeutic endoscopy [16, 16].

Acceptance of a new technology was assessed using the Van der Laan acceptance scale with a five point scale ranging from -2 to + 2, including perceptions of usefulness and satisfaction with the technology [18] (Appendix Fig. 11 2). Finally, participants answered a standardised usability and ergonomics questionnaire scored by seven-point Likert scale. (Fig. 8).

### Data analysis

Timings of tasks were reported as mean ± standard deviation. NASA-TLX scores were reported as means to one decimal place. Van der Laan’s scores were reported as means to two decimal places.

The comparisons demonstrated in the following sections were conducted using within-subjects analysis when comparing:

- Task completion time of endoscopists with conventional versus gaze-controlled endoscopy
- Task completion time of novices with conventional versus gaze-controlled endoscopy
- NASA-TLX scores of endoscopists with conventional versus gaze-controlled endoscopy
- NASA-TLX scores of novices with conventional versus gaze-controlled endoscopy

Between-subjects analysis was conducted when comparing:

- Task completion time with conventional endoscopy by endoscopists versus novices
- Task completion time with gaze-controlled endoscopy by endoscopists versus novices
- NASA-TLX scores with conventional endoscopy by endoscopists versus novices
- NASA-TLX scores with gaze-controlled endoscopy by endoscopists versus novices
- Van der Laan’s scores by endoscopists versus novices
- Ergonomics assessment scores by endoscopists versus novices

For within-subjects analysis, the Shapiro‐Wilk test for normality of the paired differences was performed, followed by paired-samples *t *test when the test was successful, and no outliers were detected. In case of non-normal distribution of the differences or the presence of outliers, the Wilcoxon signed-rank test was used.

For between-subjects analysis, the Shapiro‐Wilk test for normality of the samples was performed, followed by independent-samples *t *test when the test was successful. In case of non-normal distribution of any of the two samples, the Mann–Whitney *U* test was applied.

For all types of statistical analysis tests, a *p*-value < 0.05 was considered significant. Statistical analysis was conducted using SPSS version 25.

## Results

### Performance between groups

**Fig. 5:**
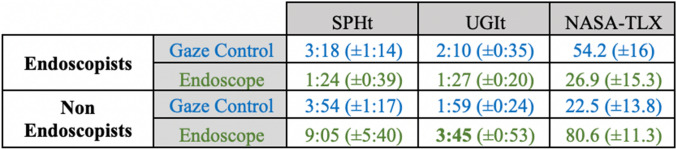
Comparison of the two modalities (gaze—hand control) for endoscopists and non-endoscopists on both setups (Spherical cavity task (SPHt), Upper Gastrointestinal tract task (UGIt)) in terms of overall task completion time (in minutes:seconds) and NASA TLX score

**Fig. 6:**
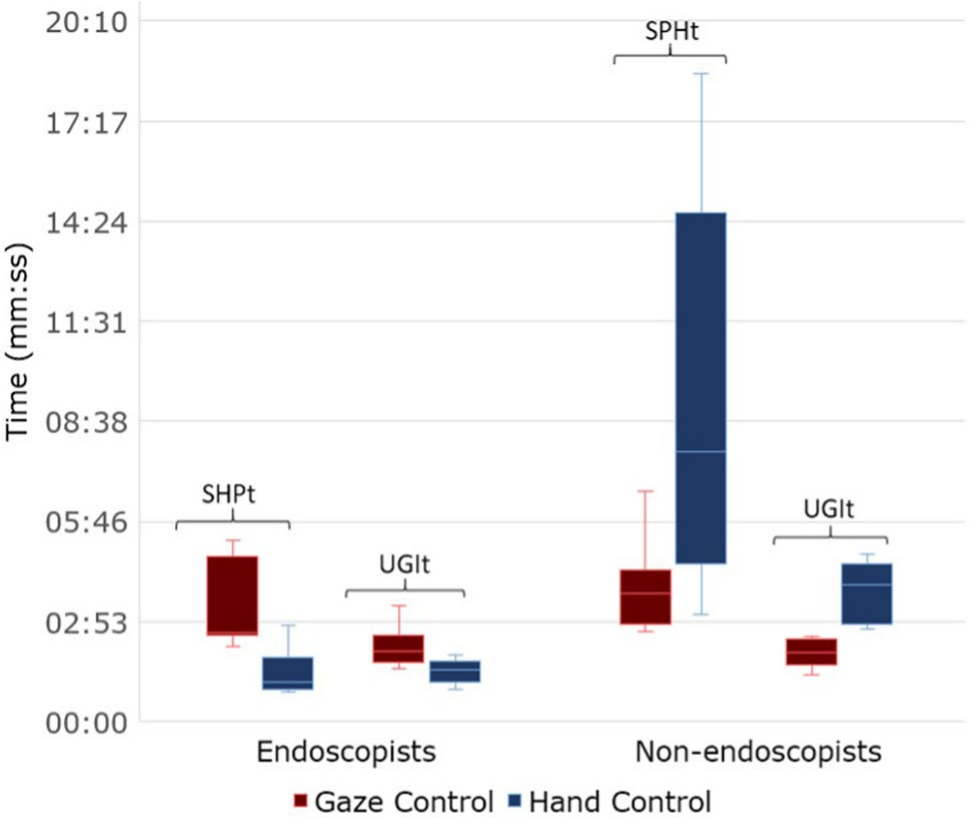
Performance comparison of the two modalities (gaze—hand control) for endoscopists and non-endoscopists on both setups (Spherical cavity task (SPHt), Upper Gastrointestinal tract task (UGIt)) in terms of overall task completion time

Both tasks had a completion rate of 100% by experienced endoscopists and by non- endoscopists. The experienced endoscopists were significantly quicker than non-endoscopists at performing each task when using conventional endoscopy (sphere task 1:24 ± 0:39 vs 9:05 ± 5:40 min, *p* = 0.006 and UGI model task 1:27 ± 0:20 versus 3:45 ± 0:53 min, *p* < 0.001) (Fig. 5), while when using gaze-control there were no significant differences between task performance (Fig. 6).

### Performance within groups

Within the participant groups, experienced endoscopists were significantly quicker at performing each task when using conventional endoscopy rather than gaze-control (Sphere task 1:24 ± 0:39 vs 3:18 ± 1:14 min, *p* = 0.002, and UGI model task 1:27 ± 0:20 vs 2:10 ± 0:35 min, *p *= 0.006).

When using gaze-control endoscopy, non-endoscopists were significantly quicker at performing each task when using gaze-control rather than conventional endoscopy (sphere task 3:54 ± 1:17 vs 9:05 ± 5:40 min, *p *= 0.012, and UGI model task 1:59 ± 0:24 vs 3:45 ± 0:53 min, *p* < 0.001).

### Task load

**Fig. 7:**
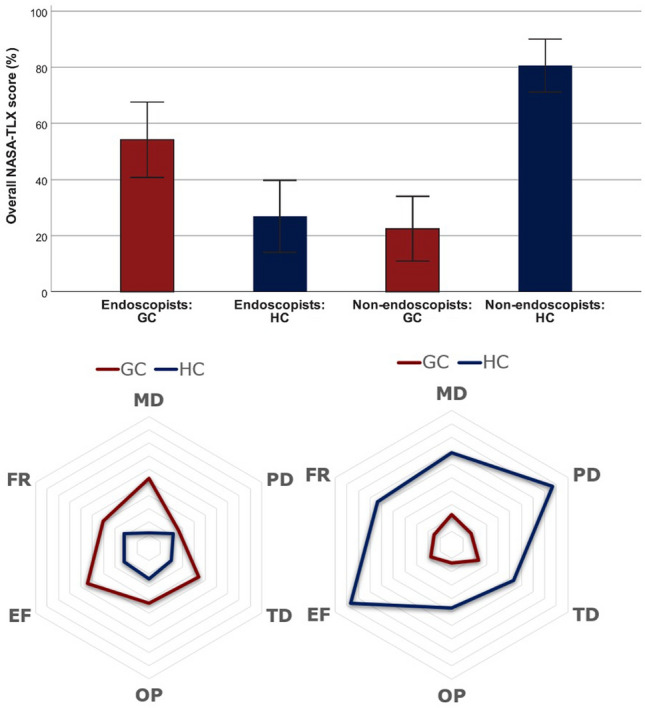
**A** Overall NASA-TLX score and analytical results (MD, PD, TD, OP, EF, FR) for **B** endoscopists and non-endoscopists. NASA-TLX values range between 0 and 100, with higher values indicating higher task load. MD: mental demand, PD: physical demand TD: temporal demand, OP—own performance, EF—, FR—frustration, HC: hand control, GC: gaze-contingent control

The NASA-TLX scores are displayed in Fig. 6. Endoscopists reported significantly higher total NASA-TLX workload scores using gaze-control versus conventional endoscopy (54.2 ± 16 vs 26.9 ± 15.3, *p* = 0.012), including significant differences for ‘mental demand’, ‘temporal demand’, ‘performance’, and ‘effort’. The inverse was true for non-endoscopists, who reported significantly higher NASA-TLX workload total scores when using conventional endoscopy versus gaze-control (80.6 ± 11.3 vs 22.5 ± 13.8, *p* < 0.001). The differences were significant across all six domains of the NASA-TLX (Fig. 7).

### Technology acceptance

**Fig. 8:**
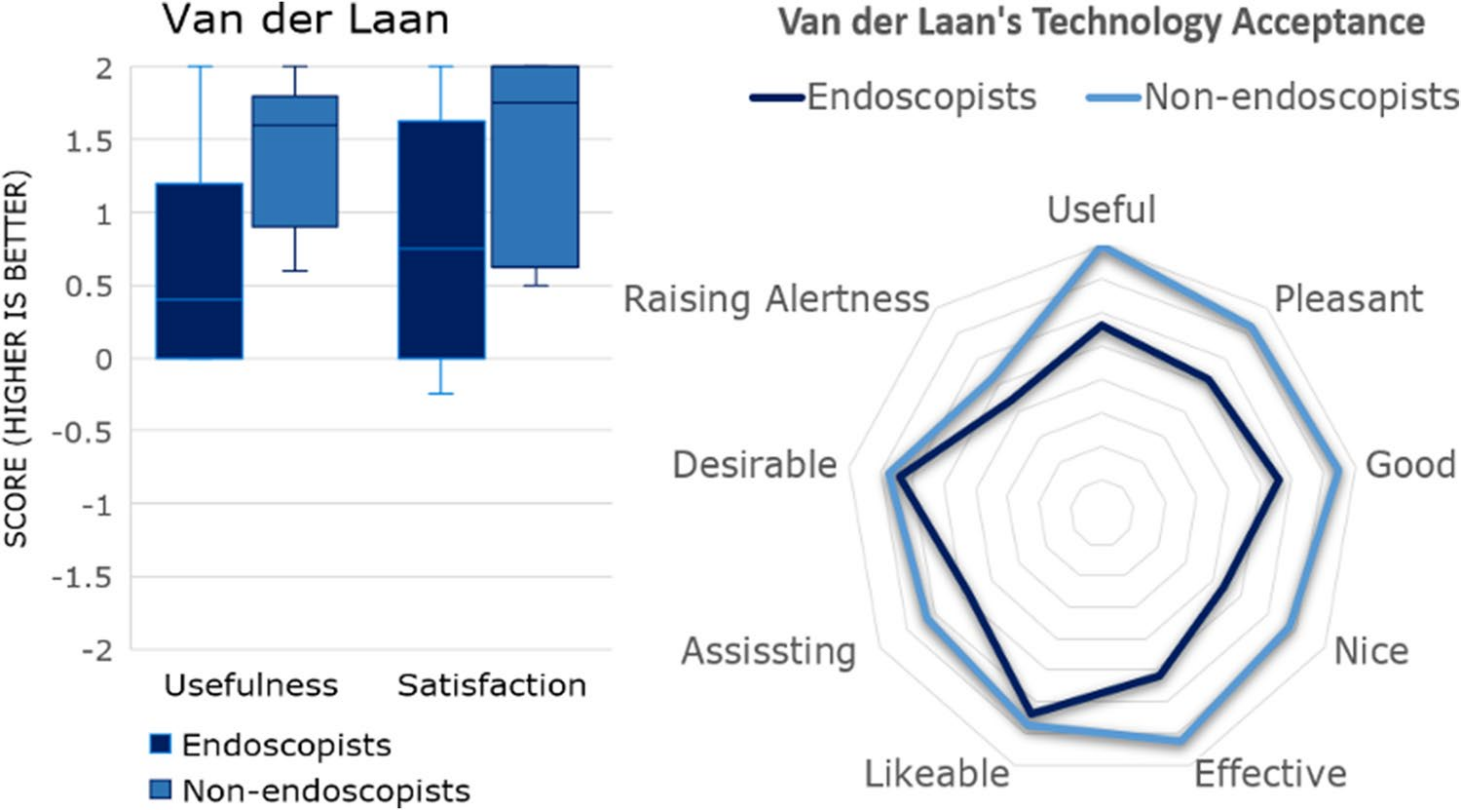
**A** Overall Van der Laan’s technology acceptance score by endoscopists and non-endoscopists and **B** analytical results. The usefulness scale derives from the average of useful/useless, good/bad, effective/superfluous, assisting/worthless, raising alertness/sleep-inducing metrics and satisfaction scale derives from pleasant/unpleasant, nice/annoying, likeable/irritating, desirable/undesirable metrics. The scale range between − 2 and + 2, with higher values indicating positive bias

Experienced and non-endoscopists reported that the gaze-control had positive ‘usefulness’ scores of 0.56 ± 0.83 and 1.43 ± 0.51 respectively using the Van der Laan scoring, displayed in Fig. 8. Usefulness and satisfaction scores showed no significant difference between non-endoscopists and experienced endoscopists. (*p *= 0.065 and *p* = 0.222 respectively). Both experienced and non-endoscopists reported positive ‘satisfaction’ scores (0.8 ± 0.87 and 1.44 ± 0.68 respectively).

### Ergonomics

Endoscopists found gaze-control to have poor ergonomics in all domains on the Likert scale other than ease of learning; being less comfortable (86%), more stressful (57%), task flow interrupting (85%), more uncomfortable for the neck (57%) strenuous for the eyes (86%) and causing fatigue (71%).

**Fig. 9:**
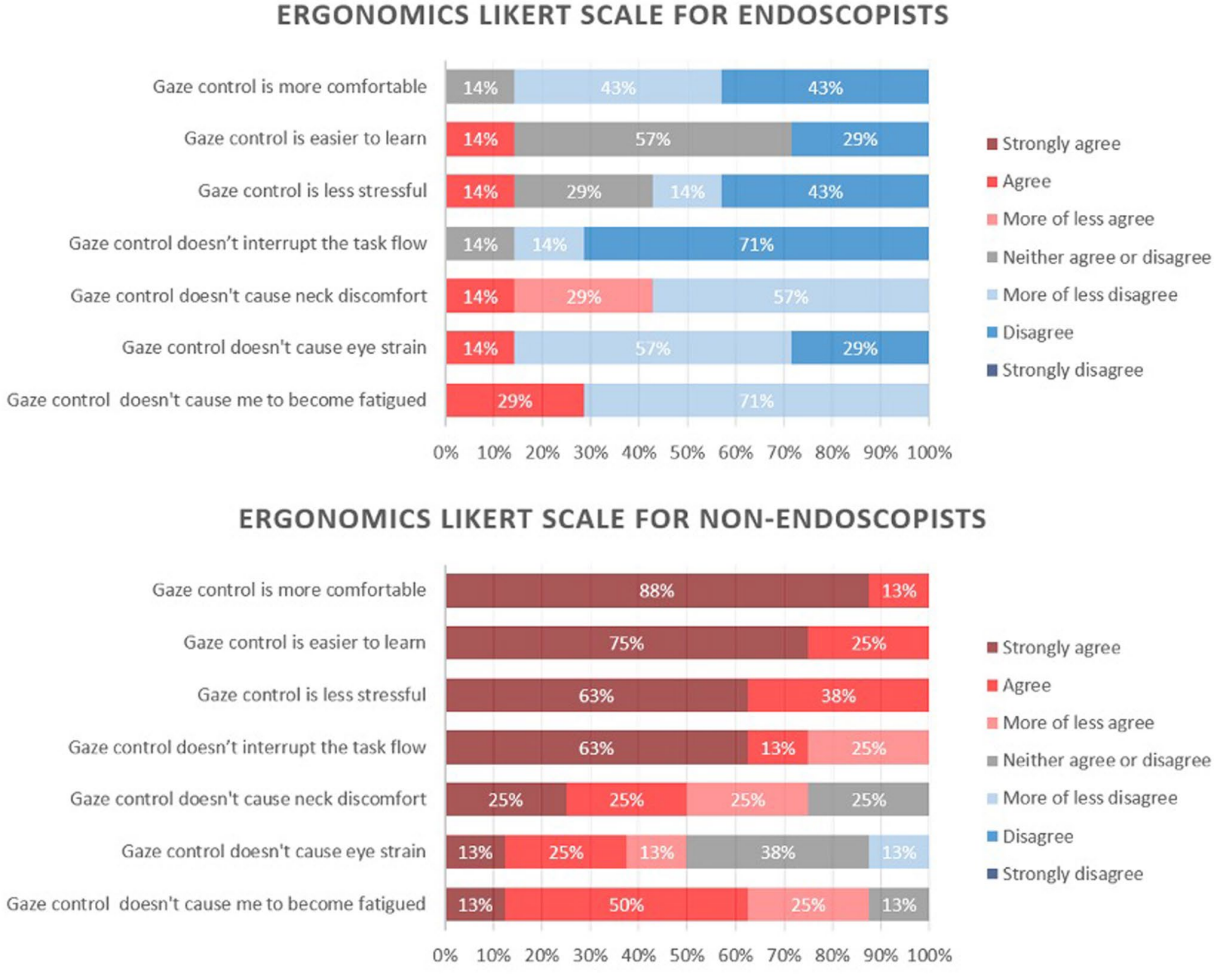
Likert scale results of ergonomics assessment for **A** endoscopists and **B** non-endoscopists

By contrast, non-endoscopists found gaze-control to have good ergonomic characteristics in all domains; being more comfortable (100%), easier to learn (100%), less stressful (100%), not task flow interrupting (100%), not uncomfortable for the neck (75%) not strenuous for eyes (51%) and not causing fatigue (88%). This is displayed in Fig. 9.

Statistical analysis confirmed significant differences between endoscopists and novices in comfort, ease of learning, stress and task flow interruptions.

## Discussion

We present the early pilot data from a fully robotised, gaze-controlled flexible endoscope platform, which allows touch-free control without loss of function. Testing this concept has demonstrated the capability of this approach to navigate a luminal cavity and display fine motor control.

The experienced endoscopists had performed many hundreds to thousands of endoscopies using conventional endoscopes. Experienced endoscopists were faster at performing the tasks using a conventional endoscope, which likely represents a familiarity with the control systems rather than a technical superiority of the traditional endoscope.

The non-endoscopists were novices to both the conventional endoscope and gaze-control system. Interestingly this group both favoured and performed quicker with the gaze-control system. This may reflect that the gaze-control is easier to learn and more intuitive than a traditional handheld and manually controlled endoscope.

### Limitations

#### Device

In its current iteration there is no substitute for the tactile feedback during conventional endoscopy. In future iterations force feedback sensors could be attached to the endoscope handles and motors and be visually represented on the screen or through haptic feedback via the joystick.

The system presented is designed specifically for the Karl-Storz gastroscope. It would be possible to incorporate a user-friendly calibration step in the future so the system could be generically applicable to endoscopes from different manufacturers and lengths of endoscope. The closed loop, gaze control method used would also nullify changes in different aged endoscopes and the changes in tensility of the steering cables.

This device was trialled in a simulated rigid environment which doesn’t account for the luminal and tissue deformation in a real patient. This group has experience in tracking for robot control, motion compensation and depth extraction [19–21]. These approaches could be incorporated into cancellation of tissue movement in future iterations.

Although eye gaze control seems feasible shared control of the scope, with a combination of hands-on and gaze control, is currently being explored.

The horizontal rail on which the endoscope is mounted has a large footprint making it less clinically practicable. Current work is underway to turn this into a coiled design with a subsequently reduced footprint.

#### Study

When looking at the individual parameters making up the NASA-TLX score, it is likely that experience will indeed affect/bias endoscopist reporting where workload may be reported relative to their experience of the conventional system [22]. The inclusion of non-endoscopists was intended to remove this training bias by assessing participants with no previous experience. This does raise the question of whether NASA-TLX is the best assessment of workload for use of novel endoscopic devices but it does appear to be an accepted method of assessment for endoscopist workload [23, 23, 23].

This study is limited in that it only compared non-endoscopists to experienced endoscopists. Further work with repeated use of each platform by non-endoscopists will help assess if the gaze-controlled platform truly has a shallower learning curve when quantitative parameters are assessed with repetition of tasks by endoscopist of varying experience (including novice endoscopists).

Lower gastrointestinal endoscopy has distinct challenges such as loop resolution and torque steering, therefore this feasibility study was not easily translatable to lower gastrointestinal applications in its current form.

## Conclusion

Endoscopic equipment has developed at great pace in recent years, allowing the introduction of ever more complex procedures facilitated by high-quality imaging as well as insertable and tip-mounted accessories. As interventions become more complex, we anticipate the need for progress in control systems, to aid the endoscopic operator. With further progress gaze-control may allow intuitive control of the endoscopic field of view and allow the operator to handle endoscopic tools without the need for multiple assistants. This could reduce cognitive burden and distraction resulting in improved resections and procedural outcomes in complex therapeutic procedures.

### Supplementary Information

Below is the link to the electronic supplementary material.

Supplementary file1 (MP4 125831 kb)

## Figures and Tables

**Fig. 10:**
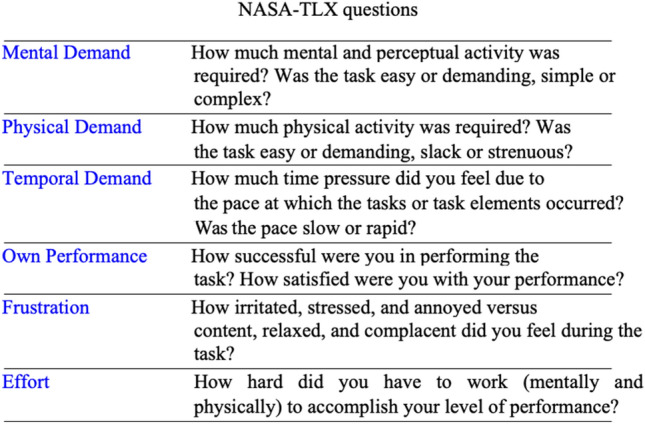
NASA-TLX [15]

**Fig. 11:**
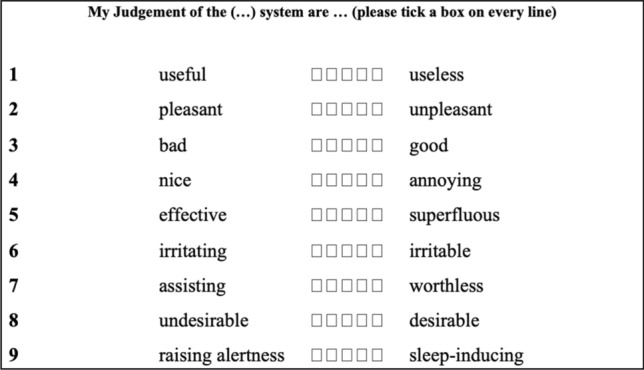
Van der Laan technology acceptance scale [18]

## Appendix 1

See Fig. 10.

## Appendix 2

See Fig. 11.
